# The Association between Ponticulus Posticus and Dental Agenesis: A Retrospective Study

**DOI:** 10.2174/1874210601812010510

**Published:** 2018-07-31

**Authors:** Alessandra Putrino, Rosa Maria Leonardi, Ersilia Barbato, Gabriella Galluccio

**Affiliations:** 1 Department of Oral and Maxillofacial Sciences, University “Sapienza” of Rome, Roma, Italy; 2 Department of Medical and Surgical Sciences, II Dental Unity, University of Catania, Catania, Italy

**Keywords:** Dental anomalies, Ponticulus Posticus (PP), Dental Agenesis (DA), Atlantooccipital Ligament (AOL), Oligodontia, Hypodontia, Anodontia

## Abstract

**Objective:**

Neural tube defects may increase the risk of an abnormal development of skull, vertebral column and teeth formation, including dental agenesis in non syndromic patients. The association between the presence of a congenital Dental Agenesis (DA) and the Atlantooccipital Ligament (AOL) calcification, known as “Ponticulus Posticus” (PP), as possible links can be investigated.

**Design:**

After a systematic review of the scientific literature on this topic, two independent examiners assessed the AOL calcification in lateral cephalograms of 350 non syndromic patients(7-21 years old). The results were compared with a control group (non syndromic patients, without congenital missing teeth).

**Results:**

The 16.3% of the population studied by cephalometric analysis revealed a prevalence rate of PP (both complete and partial) with a slight male predominance is seen, not statistically significant (χ square test = 0.09; *p*= 0.76). In both sexes complete PP is more observed. In the patients affected by DA the frequency of PP is the 66.6% (both complete than partial). The χ square test with Yates correction showed a significative difference(χ= 66.20; *p* value= 0.00) between PP in patients with DA compared to not affected by DA.

**Conclusions:**

PP is not an uncommon anomaly. Since orofacial pain like migraine and other symptoms are often associated to PP, during routine radiographic examination, if detected, it should be documented in patients’ health record and with symptoms, further investigation should be sought for. These findings encourage to think there’s an association between DA in non syndromic patients and neuro-crestal cells defects.

## 1 INTRODUCTION

Many of the common dental anomalies affecting the human dentition during odontogenesis are interrelated to the embryologic processes involved in facial, jaws and vertebral column development. The related molecular and genetic factors control these events are well documented in literature. The most common congenital dental anomaly is the absence of teeth, it can affect permanent and deciduous dentition.

### 1.1 Dental Agenesis

Excluding third molars, when the absence of one to six teeth is observed it’s defined “hypodontia”, up to six teeth is “oligodontia”, while the absence of all teeth is “anodontia” [1-3]. Hypodontia is usually associated with other oral and dental and altered craniofacial growth [4]. Dental agenesis can occur in non-syndromic population [5, 6], like familiar form, or be part of a multi-symptomatic syndrome [7]. In the majority of cases dental agenesis has genetic origins but also dental traumas, infections like rubella, chemo or radiotherapy, disturbances in local innervations, environmental situations, may influence and lead to the congenital absence of teeth [2, 7].

During the daily clinical evaluation, especially in young and child patients, it’s important to know about the correlation between dental agenesis and genetic mutations [8]. A wide number of genes are involved in tooth morphogenesis [9-11]. The primitive epithelium covers the stomodeum and the neural crest cells regulate the process of tooth development [12-14].

### 1.2 Neural Crest Cells

In many parts of the skull and the mouth, teeth included, there are tracks of the neural crest cells migrated from the original neural tube [15, 16]. Neural crest constitutes a pluripotential mesenchyme that is the source of diverse tissue types: neural, glial, skeletal, connective, pigment, and secretory. In the head region, neural crest forms a major port ion of the skull, maxilla, mandible, auditory ossicles, hyoid bone, larynx, dental tissues (dentin, cementum), the periodontal ligament, the alveolar bone and the vertebral column [17-20]. Publications on adult populations confirm the association between malocclusions, skeletal deviations in both the jaws, skull base and cervical spine anomalies that can be prenatally observed [21-23].

### 1.3 Ponticulus Posticus

One of the skeletal column anomaly related to neural crest cells activity during foetal development is the ossification/calcification of the posterior atlanto-occipital ligament, known as “ponticulus posticus”(latin expression for “little posterior bridge”), which describes an *“anomalous malformed bony bridge between the posterior portion of the superior articular process and the posterolateral portion of the superior margin of the posterior arch of the first cervical vertebra (atlas)”* [24]. In its presence, “*there is occlusion of the vertebral artery and patients with ponticulus posticus often display symptoms of vertebrobasilar insufficiency such as headache, vertigo and diplopia”* [25].

The prevalence of ponticulus posticus has been reported to be between 5.1% and 37.8% in the Western population and a female predominance has been reported in the literature [25]. The ponticulus posticus radiologic evidence has been documented and classified as related to different levels of ossification. Three types of ponticulus posticus can be detected on cephalograms and lateral radiographs [26, 27]:

- Full type: A complete bony ring.
- Incomplete type: An uncomplete bony ring.
- Calcified type: A linear or amorphous calcification.

A research documented the presence of a mildest calcification in the sampled population more similar to a small spicule [28]. This retrospective study aims to search a significative involvement of neural crest cells, under genetic control, in patients affected by non syndromic hypodontia and by alterations of skeletal structures (presence of ponticulus posticus) using radiographic examinations.

## 2 MATERIALS AND METHODS

Before to collect the clinical data about patients involved in the study, a systematic review of the scientific literature published on PubMed, Lilacs and Scopus data base has been done. The key words used were: “atlanto-occipital ligament”, “ponticulus posticus”, “neural crest cells”, “orthodontic”. No restrictions of time and languages have been fixed. The results have been filtered and valued following our eligibility criteria and then organized following the PRISMA method [29]. It’s a custom to ask and obtain informed consent for the scientific aims at the time patients access to orthodontic treatment in our unit.

We followed these inclusion criteria:

- Abstracts available;
- Radiographic evaluation on lateral cephalograms;
- Non-syndromic population;
- Related to the aim of the study (orthodontic patients, research of ponticulus posticus anomaly);

The study was performed in December 2014 at the Unit of Orthodontics at University “La Sapienza” in Rome. Lateral cephalograms and panoramic radiographs were collected from the archives and examined for ponticulus posticus and dental agenesis. Patients with history of cleft lip palate, facial/dental traumas and vertebral surgery for the treatment of cervical spine were excluded. The sampled population of patients was Caucasian and African. In case of poor visualization or overlapping of the interested structures cephalograms have been not considered in this study.

The sampled population counted a total of 350 subjects (160 males and 190 females) with a mean age of 13 (Table **1**).

We considered the initial lateral cephalograms and orthopantomographs detecting the data at the early stage of clinical-radiographic observation. In those patients with dental agenesis but no ponticulus posticus, we considered also cephalograms subsequent to the first, if requested for orthodontic reasons, to exclude or confirm the presence of ponticulus posticus. In patients with a partial or not clear presence of ponticulus posticus, we also considered eventual next cephalograms, if attached to the clinical documentation. The radiographs were viewed on a flat monitor (LCD,Samsung) in JPEG format. The cephalograms on traditional films have been scanned (Epson Scanner) and saved as .jpeg files to zoom in/adjust in contrast the image and allow a better evaluation of the occipital-atlas vertebra relations. The inspection of each radiograph to detect the presence of any type of ponticulus posticus has been performed by two independent operators (AP and GG). To eliminate possible errors, 50 cephalograms choosed randomly have been reexamined separately by the same operators in January 2015 (K test=100). All collected data were recorded on virtual sheets in Microsoft Excel 2007. Functions for statistical analysis have been properly selected. The χ square test with Yates correction was used to measure the association between ponticulus posticus and gender.

## 3 RESULTS

**Fig. (1):**
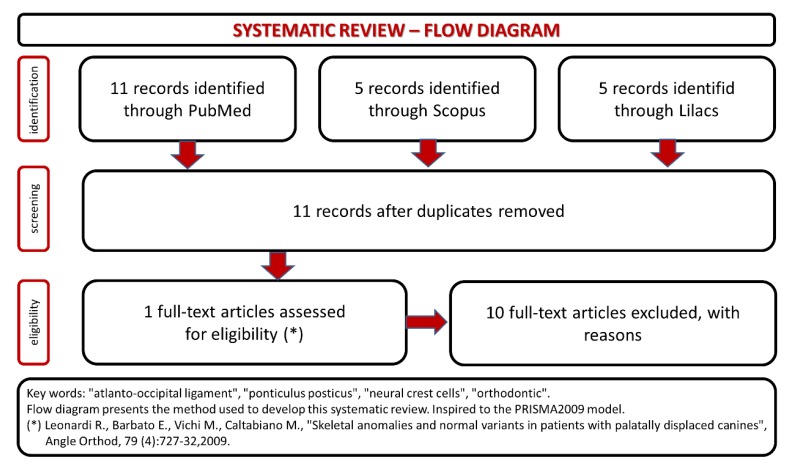
Flow chart illustrates the pathway followed for a systematic review of literature about the aim of this study.

The systematic review of the literature results have been summarized in the flow chart (Fig. **1**). There’s a poor production of articles related this topic, and even if all the papers considered in the study used cephalograms like diagnostic tool to find ponticulus posticus in patients, only one study searches a positive association between the presence of ponticulus posticus and dental/orthodontic evaluations [30]. The others are useful to document the incidence of ponticulus posticus in the population.

**Fig. (2):**
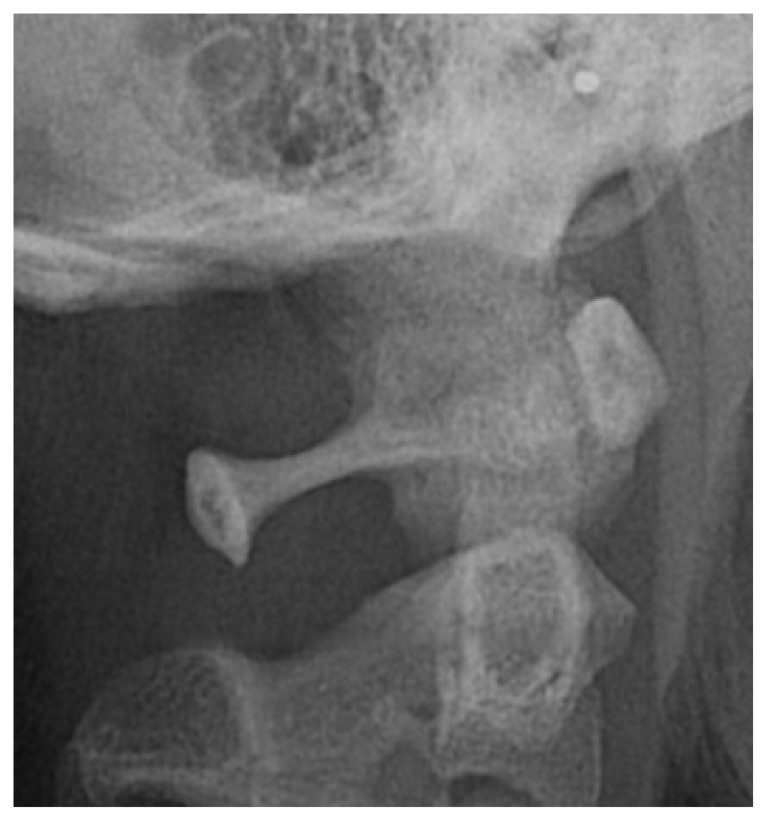
Detail of a normal atlas as viewed on lateral cephalogram.

The direct inspection of the 350 lateral cephalograms (Fig. **2**) has lead to find a prevalence rate of 32,95% for both the types complete and partial ponticulus (Table **2**), the presence of calcified type has never been observed. A slight male predominance without statistical relevance has been observed (χ square test = 0.09; *p* value= 0.76) (Table **3**).

No disagreement intra-observer and between the two observers at the first and the second random examination has been found.

**Fig. (3):**
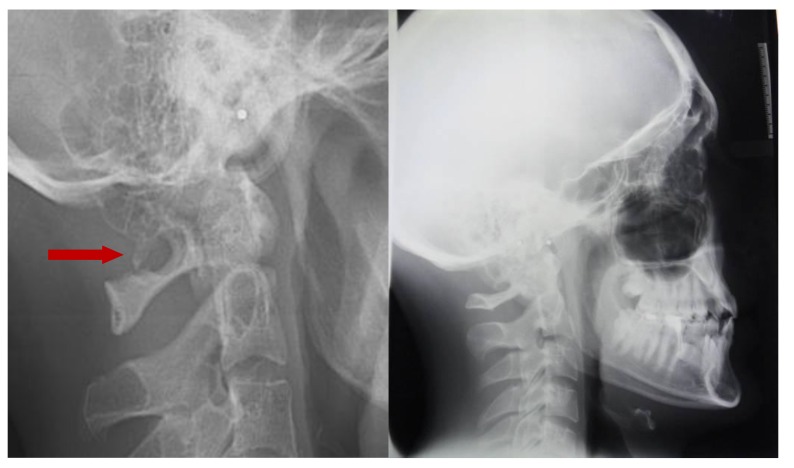
Detail of cephalogram showing partial type ponticulus posticus on the left, and cephalogram showing a partial ponticulus posticus in a patient with agenesis of 35.

**Fig. (4):**
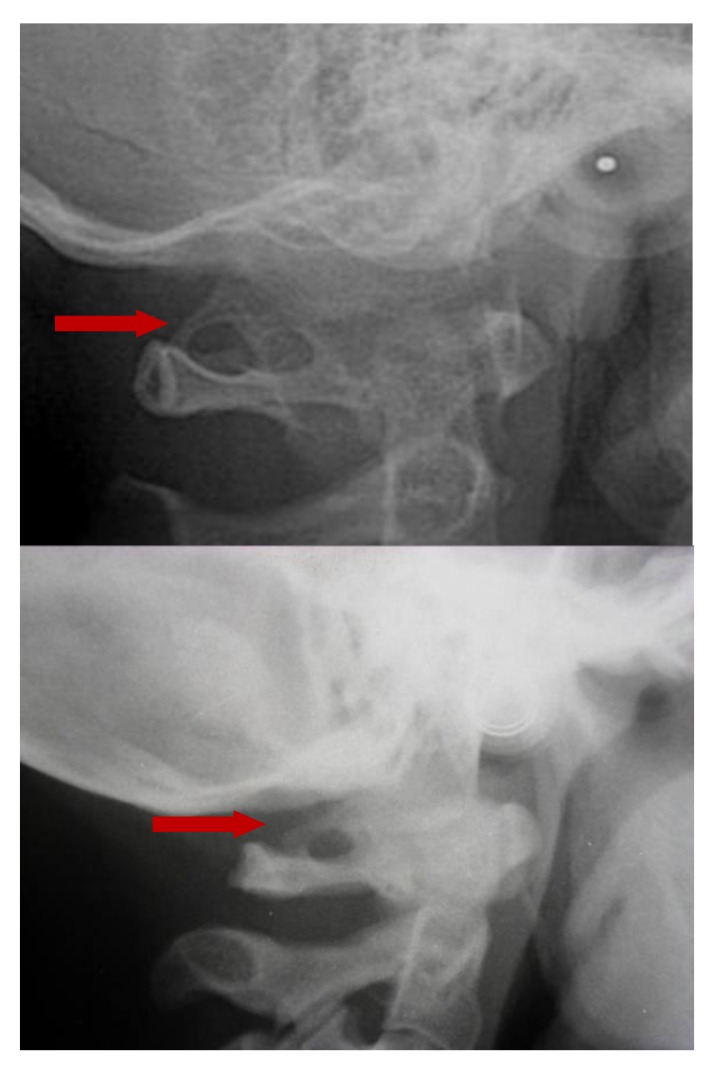
Detail of cephalogram showing a complete ponticulus posticus and a full cephalogram of patient with agenesis of 45 and complete ponticulus posticus.

The prevalence rate of dental agenesis in the studied population is around 12%. In order of frequency the tooth more often missing for agenesis is the upper lateral incisor, followed by second and first premolars, more often mandibular than maxillar. In the female like in the male patients is more observed complete ponticulus posticus than partial. In the patients affected by dental agenesis the frequency of ponticulus posticus (Fig. **3** and **4**) is the 66.6% (both complete than partial), it’s not observable in the 33.3% of them. A percentage around 11% represents patients non affected by dental agenesis but with the evidence of a ponticulus posticus in their cephalograms. Three of them had the inclusion of canines, one had an history of relatives with non syndromic agenesis. Four cephalograms have been excluded for a not quality of the radiographic image. The measurement of prevalence of ponticulus posticus in patients with dental agenesis compared to the number of observations in patients not affected by dental agenesis has been calculated statistically by the χ square test with Yates correction (Table **4**). The results show a significative difference (χ= 66.20; *p*-value= 0.00) (Table **5**). Anamnestic data available in the clinical diary attached to the radiographs of patients with ponticulus posticus in the archive don’t describe migraines, column pains or whatever symptom related to the cervical ponticulus posticus, except one with dental agenesis too. The 70% of patients with ponticulus posticus not affected by dental agenesis showed to be a II class (skeletal).

## 4 DISCUSSION

Different studies esteemed the rate of prevalence ponticulus posticus around 5.1% and 37.8% [31, 32]. The prevalence of the complete type has been reported to be 2.6% and 14.3% like radiographic finding and around 3.4% and 15% in osteological researches [33]. A study on cephalograms of Peruvian patients [34] documented a frequency of complete ponticulus posticus in 19.79%, partial on 11.08%. In the article documents our research the complete type of ponticulus posticus has been found in 19% of the patients. A female prevalence has been reported in literature [32,35,36, 24] , but our study results show a different trend, even if it’s proved to be not statistically significant. Indian orthodontic patients [25] showed a prevalence of complete ponticulus posticus (4.3%) lower the rate found in this study (20.92%). The same study compared to our results showed opposite trends for gender prevalence: male (5.33%) for them, over female (3.76%) for us. In the study done by Gupta *et al*, complete ponticulus posticus, recorded in 11.1% of sampled patients, is seen in 2.8% of males and 3.1% of females [24]. In our study we found a significative evidence of ponticulus posticus in patients with dental agenesis without predominance of partial or complete posticus related to age or gender of these patients. In a study Leonardi R. *et al.* (2009) found a positive association of ponticulus posticus in patients with impacted canines [30]. The age doesn’t seem to influence the level of ossification of atlanto-occipital ligament, since we observed complete ponticulus posticus in very young children (7-8 years old) and at the same time partial ponticulus posticus in older children, adolescents and young adults too. We can’t admit ponticulus posticus evolve necessarily from an initial to a complete type.

The prevalence of ponticulus posticus is strictly influenced by the quality, diagnostic power and level of radiographic images like documented in a study performed on Korean population [36] where, on the same population studied, the prevalence of this anomaly increased from 6.95% using a plain radiograph to 15.5% using a 3D CT scan. Also in another study [37] the prevalence documented was higher than in our results, around 45.9%, but this difference can be attributed to the numbers of the patients sampled, double than ours. There are no reports on whether the ponticulus posticus represents the result of bone formation by heterotopic ossification or a mineralized sof tissue by ectopic calcification [38]. Tubbs *et al*. (2007), noted that the no ossification centers can be found in ligaments involved in ponticulus posticus structure [39]. Crowe (1986) found ponticulus posticus in patients younger than 15 years old and those findings suggest that the ponticulus posticus is not a calcified ligament and not related to ligamental stress , because ligamentous calcification occurs years following the final formation of bone, and then following prolonged stress [40]. Hong *et al*. (2008) found statistically significant differences between the partial and complete ponticulus posticus, and suggested that the formation of a ponticulus posticus would appear to be similar to osteophyte formation, a condition also related to age [41]. The population for the present study was predominantly under 18 years because of being an orthodontic population and the differences found for the age variable are in agreement with the findings reported by Tubbs *et al*., Crowe and Hong *et al* [39-41]. In the study documented by Paraskevas *et al*. (2005) a progressive calcification has been reported [42]. The importance of early identification of this anomaly on routine lateral cephalograms asked for orthodontic diagnosis is enhanced by the correlation with its presence and the diagnosis of popular disturbs like migraine [43], detected in the 15.8% of cases sampled with ponticulus posticus, the association with TMJ disorders [44, 45], or in the identification of more rare clinical pictures like in the nevoid cell carcinoma syndrome [46]. Especially in all of those cases of orofacial pain disorders, migraines and chronic tension type headaches, the patient giving evidence is essential bearing in mind that complications related to this vertebral anomaly can lead to the need of cervical spine surgery [47,48, 35]. The history of symptoms is absent in our study, since it has been designed like a retrospective research and data collected by patients’ clinical diaries are lack of these informations able to further prove those linkings documented in literature and cited above but we can’t exclude our patients are asymptomatic.

## CONCLUSION

This study showed that ponticulus posticus is not an uncommon or definetely rare anomaly in the Italian population, and its positive relation with dental anomalies, like dental agenesis, encourage us to believe in the power and importance of basic investigation on cephalograms. When detected, in our routine radiographic examination, it should be documented in patients’ health records, especially in the symptomatic ones. The relationship between the prenatal development of the cranium and some related structures like teeth and column are under the control of same genes and this process, with the possible abnormal activities and results, can’t be considered a field of interest confined to genetics. Our study indeed showed how this relationship can influence our orthodontic diagnosis making our treatment planning more complete and our daily activity more precious and helpful in the management of our patients’ health beyond our field of intervention and practice just being able to read the not so hidden findings of our regular investigation tools.

## Figures and Tables

**Table 1:** Distribution of patients sampled for the study.

**Sex**	**Number of Patients**	**Age Range**	**Mean Age**
Males	160	7-21 years	13.4
Females	190	7-21 years	12.5
Total	350	7-21 years	12.9

**Table 2:** Prevalence of two types of ponticulus posticus (linear never detected) in males and females patients selected in this study.s

**Type**	**Males**	**Females**
**Complete**	20 (12.5%)	16 (8.42%)
**Partial**	10 (6.25%)	11 (5.78%)
**Calcified/Linear**	0 (0%)	0 (0%)
**Total**	30 (18.75%)	27 (14.2%)

**Table 3:** χ square test results even with Yates correction show no differences statistically significant about the distribution of ponticulus posticus in the gender male and female.

**Chi- Square Test**					**0,34**	→ Non significative difference		
-	-	-	-	*p:*	0,5627	-	-	-
**Chi – Square Test (with Yates correction):**					**0,09**	→ Non significative difference		
-	-	-	-	*p:*	0,7612	-	-	-

**Table 4:** Distribution of radiographic evidence of ponticulus posticus in patients with and without dental agenesis.

**Radiographic Evidence**	**Patients with Dental Agenesis**	**Patient without Dental Agenesis**	**Total**
**Ponticulus Posticus**	28 (8%)	39 (11%)	67 (19%)
**No Ponticulus Posticus**	14 (4%)	269 (77%)	283 (81%)
**Total**	42 (12%)	308 (88%)	350 (100%)

**Table 5:** χ square test results even with Yates correction show differences statistically significant about the distribution of ponticulus posticus in the patients with and without dental agenesis.

**Chi-Square Test**					**69,64**	→ Significative difference (prob. 1%)			
-	-	-	-	*p:*	0,0000	-	-	-	-
**Chi – Square Test (with Yates correction):**					**66,20**	→ Significative difference (prob. 1%)			
-	-	-	-	*p:*	0,0000	-	-	-	-
